# Systematic Analysis of the Expression and Prognosis of Fcγ Receptors in Clear Cell Renal Cell Carcinoma

**DOI:** 10.3389/fonc.2022.755936

**Published:** 2022-03-17

**Authors:** Wenyuan Nie, Yong Yao, Benjun Luo, Jiyin Zhu, Shaocheng Li, Xiaoteng Yang, Tao Luo, Wei Liu, Shibing Yan

**Affiliations:** Department of Urology, Medical Center Hospital of Qionglai City, Chengdu, China

**Keywords:** clear cell renal cell carcinoma, Fcγ receptor, prognostic value, bioinformatics analysis, tumor microenvironment

## Abstract

**Background:**

Clear cell renal cell carcinoma (ccRCC) remains a common malignancy in the urinary system. Although dramatic progress was made in multimodal therapies, the improvement of its prognosis continues to be unsatisfactory. The antibody-binding crystallizable fragment (Fc) γ receptors (FcγRs) are expressed on the surface of leukocytes, to mediate antibody-induced cell-mediated anti-tumor responses when tumor-reactive antibodies are present. FcγRs have been studied extensively in immune cells, but rarely in cancer cells.

**Methods:**

ONCOMINE, UALCAN, GEPIA, TIMER, TISIDB, Kaplan–Meier Plotter, SurvivalMeth, and STRING databases were utilized in this study.

**Results:**

Transcriptional levels of FcγRs were upregulated in patients with ccRCC. There was a noticeable correlation between the over expressions of FCGR1A/B/C, FCGR2A, and clinical cancer stages/tumor grade in ccRCC patients. Besides, higher transcription levels of FcγRs were found to be associated with poor overall survival (OS) in ccRCC patients. Further, high DNA methylation levels of FcγRs were also observed in ccRCC patients, and higher DNA methylation levels of FcγRs were associated with shorter OS. Moreover, we also found that the expression of FcγRs was significantly correlated with immune infiltrates, namely, immune cells (NK, macrophages, Treg, cells) and immunoinhibitor (IL-10, TGFB1, and CTLA-4).

**Conclusions:**

Our study demonstrated that high DNA methylation levels of FcγRs lead to their low mRNA, protein levels, and poor prognosis in ccRCC patients, which may provide new insights into the choice of immunotherapy targets and prognostic biomarkers.

## Introduction

Renal cell carcinoma (RCC) is one of the most common malignancies of the urinary system, which accounts for 3–5% of all new cases of cancer worldwide (1). Clear cell RCC (ccRCC) is the main type of RCC that accounts for 75–82% of the incidence (2). Although immunotherapy strategies of metastatic RCC have been partially improved in recent decades, namely, cytokines, monoclonal antibodies, immuno checkpoint inhibitors(ICI), and chimeric antigen receptor (CAR) modified immune cells therapy, the improvement in the clinical results of the patient still remained unsatisfactory duo to the multiple immune escape mechanisms of kidney cancer (3).

The family of Fc receptors for IgG (FcγRs) are membrane-bound glycoproteins, expressed by several types of circulating and tissue-resident leukocytes (4, 5), which act as a bridge between specific antibodies and effector cell functions to make innate immunity and adaptive immunity closely related (6). To date, three different classes of FcγRs, known as FcγRI, FcγRII, and FcγRIII, have fully recognized in humans (7). FcγRI, which exists on the membrane surface of monocytes and macrophages, has a high affinity with IgG (8). Three genes encoding FcγRI have been identified, which are *FCGR1A*, *FCGR1B*, and *FCGR1C*, whereas only *FCGR1A* expresses the functional FcγRI, *FCGR1B/C* are duplicated pseudogenes of *FCGR1A* (9, 10). Contrary to FcγRI, FcγRII, and FcγRIII exhibit low affinity for monomeric IgG, but they are capable of binding IgG–antigen complexes through high avidity, multimeric interactions (11). Three different FcγRII have been identified, FcγRIIa, FcγRIIb, and FcγRIIc are encoded by *FCGR2A*, *FCGR2B*, and *FCGR2C* respectively and mainly expressing on B lymphocytes, granulocytes, monocytes, macrophages, and dendritic cells (12, 13). FcγRIIb is the sole inhibitory FcγR which can counterbalance the signaling activity of the activating FcγRs. Two classes of FcγRIII (FcγRIIIa and FcγRIIIb) are encoded by the *FCGR3A* and *FCGR3B* genes. FcγRIIIa is widely expressed by macrophages, NK cells, and monocyte subsets, while FcγRIIIb expression is restricted to neutrophils (14, 15).

FcγRs are involved in anti-tumor immunity in the following ways. 1. FcγRs expressed by natural killer (NK) cells and macrophages engage with antibody (IgG), triggering antibody-dependent cellular cytotoxicity (ADCC) of tumor cells (16, 17); 2. Anti-tumor antibodies bind to phagocytic surface FcγRs to enhance the phagocytic function of phagocytosis (18). 3. Anti-tumor antibodies can bind to the corresponding tumor antigen to form an immune complex, where the IgG FC segment can bind to the FcγRs on the APC surface, thus enriching the antigen, facilitating the APC presentation of tumor antigens to T cells (19).

In the past few years, polymorphisms in some members of the FcγRs have been reported in studies which lead to a different response to monoclonal antibodies in cancer (20), whereas abnormal expression of FcγRs in cancer has not been reported yet. In this present study, bioinformatics was performed initially to address this problem by analyzing the expression, DNA methylation, and prognosis of FcγRs and their relations with individual cancer stages and tumor grade in ccRCC patients. Furthermore, we also analyzed the predicted functions and pathways of FcγRs and their 88 co-expression genes.

## Materials and Methods

### Ethics Statement

The study has been admitted by the Institutional Review Board of the Medical Central Hospital of Qionglai. All written informed consent had already been obtained since all the data were retrieved from the online databases.

### ONCOMINE Database

ONCOMINE is a publicly accessible online genome-wide expression analysis platform, covering 715 datasets and 86,733 samples of cancer (21). ONCOMINE was utilized to analyze expression differences of the FcγRs gene family in multiple tumor tissues and the corresponding adjacent normal tissues. The threshold was determined according to the following values: p-value of 0.001, fold change of 1.5, and gene ranking the top 10%. In this study, the cell color is determined by the best gene rank percentile for the analysis within the cell, and the Student’s t-test was applied to generate a p-value.

### UALCAN

UALCAN is a comprehensive and interactive web resource for analyzing cancer OMICS data (TCGA, MET500, and CPTAC) (22). In our study, UALCAN was used to illustrate the distinct expression levels of tumor and normal tissues of ccRCC. Student’s t-test was used to generate a p-value and the p-value cutoff was 0.05.

### GEPIA

Gene Expression Profiling Interactive Analysis (GEPIA) is a newly developed interactive platform for elaborating the RNA sequencing expression data of 9,736 tumors and 8,587 normal samples from the TCGA and the Genotype-tissue Expression dataset, utilizing a standard processing pipeline (23). In this study, GEPIA was used to compare the association with cancer type staging of eight FcγRs members. The Student’s t-test was used to generate a p-value and the p-value cutoff was 0.05.

### TIMER2.0

TIMER is a comprehensive resource for systematical analysis of immune infiltrates across diverse cancer types. The 2.0 version of the webserver provides abundances of immune infiltrates estimated by multiple immune deconvolution methods, and allows users to generate high-quality figures dynamically to explore tumor immunological, clinical, and genomic features comprehensively (TIMER2.0 for analysis of tumor-infiltrating immune cells). In this study, we used TIMER2.0 to assess the correlation between FcγRs expression levels and immune cell infiltration and to assess the correlation between clinical outcomes and immune cell infiltration and FcγRs expression.

### TISIDB

TISIDB is a web portal for tumor and immune system interaction, and a valuable resource for cancer immunology research and therapy, which integrates multiple heterogeneous data types (TISIDB: an integrated repository portal for tumor-immune system interactions). In this study, we used TISIDB to assess the correlation between FcγRs mRNA expression levels and immunoinhibitors expression levels or cancer grade of ccRCC.

### Kaplan–Meier Plotter

The Kaplan–Meier plotter is an online database to assess the effect of gene expression on survival in 21 cancer types (24). We used this online tool to evaluate the prognostic value of FcγRs mRNA levels in ccRCC patients. The overall survival (OS) and recurrence-free survival (RFS) of patients were analyzed with a 50% (Median) cutoff for both low and high expression groups. The statically significant difference was considered when a p-value is <0.05. Information on the number of patients, median values of mRNA expression, 95% confidence interval (CI), hazard ratio (HR), and P-value can be found on the Kaplan–Meier plotter web page.

### Multivariate Regression Analysis of ccRCC Data in The Cancer Genome Atlas (TCGA) Database

We have downloaded RNA-sequencing, clinical, pathological, and follow-up data of 603 ccRCC patients from the TCGA-KIRC dataset. A total of 484 cases with complete data were screened out for multivariate regression analysis.

### SurvivalMeth

SurvivalMeth is a web server to investigate the effect of DNA methylation-related functional elements on prognosis, and multiple kinds of commonly used functional elements associated with DNA methylation are considered (25). The frequently used data from the TCGA, the CCLE, and the GEO were prestored into SurvivalMeth, namely, 81 DNA methylation profiles in 13,371 samples across 36 cancers, covering more than 480,000 DNA methylation sites locating in 19,000 coding genes, 1,689,653 super enhancers, 1,304,902 CTCF binding regions, 77,634 repeat elements and multiple functional elements such as CpG island, shore, shelf, promoter, gene body, exon, etc.

### STRING

STRING is a database of known and predicted protein–protein direct (physical) and indirect (functional) interactions (26). The protein–protein interactions (PPI) network of FcγRs co-expressed genes was visualized using the online tool of STRING with the species setting to *Homo sapiens* and a combined score of >0.7 was considered statistically significant. The nodes meant proteins; the edges meant the interaction of proteins and we hide disconnected nodes in the network.

### DAVID

Functions of FcγRs and 88 co-expression genes significantly were analyzed by the Gene Ontology (GO) and the Kyoto Encyclopedia of Genes and Genomes (KEGG) in the Database for Annotation, Visualization, and Integrated Discovery (DAVID) (24). Gene ontology analyses focus on three domains: biological processes (BP), cellular components (CC), and molecular functions (MF), and such analyses are commonly used to predict the functional roles of FcγRs mutations and 80 genes significantly associated with FcγRs mutations, while the KEGG analysis can define the pathways related to the FcγRs mutations and 80 co-expressed genes associated with FcγRs mutations. Only terms with p-value of <0.05 were considered as significant.

## Results

### Aberrant Expression of FcγRs in Patients With ccRCC

**Figure 1:**
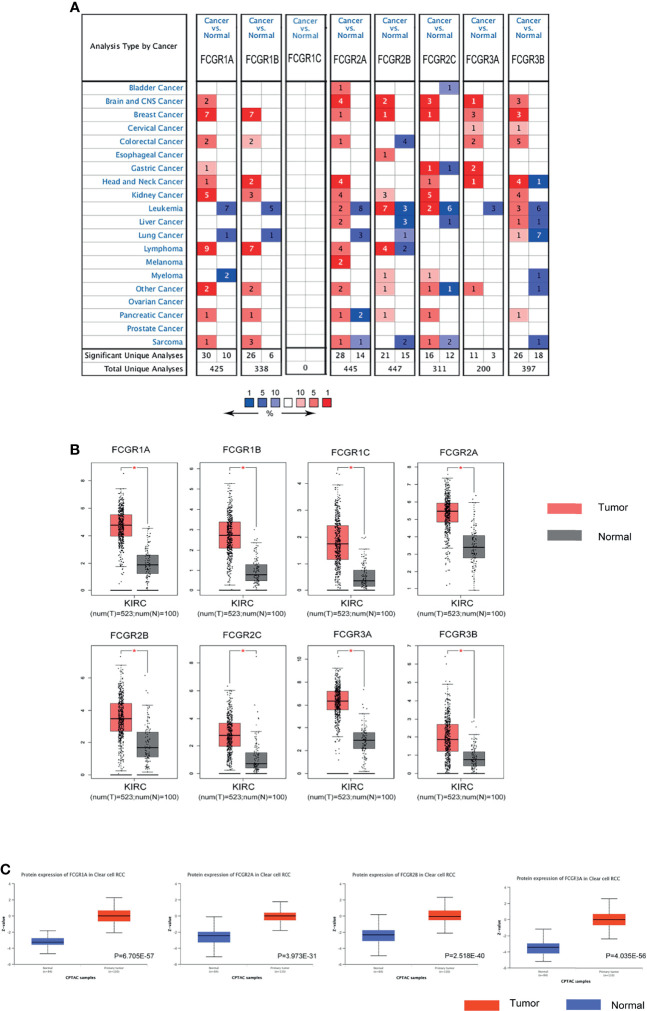
The expression of FCγRs in ccRCC. **(A)** The figure shows the numbers of datasets with statistically significant mRNA upregulation (red) or downregulated expression (blue) of FCγRs. Student’s t-test was used to compare the different mRNA levels. Cutoff of p-value and fold change were as following: p-value: 0.01, fold change: 2, gene rank: 10%, data type: mRNA. **(B)** The mRNA expression of different FCγRs in ccRCC tissues and adjacent normal tissues (GEPIA). All the FCγRs mRNA expressions were found to be upregulated in ccRCC compared to normal samples. *p <0.01. **(C)** The protein levels of *FCGR1A*, *FCGR2A/B*, and *FCGR3A* were found to be upregulated in ccRCC tissues compared to normal tissues (UALCAN).

Differential mRNA expression levels of FcγRs were profiled in tumor and adjacent normal tissues of multiple cancer types using Oncomine platform. mRNA levels of FcγR family were remarkably upregulated in four cancer types, namely, brain and CNS, breast, head and neck colorectal and kidney, while mRNA levels of FcγRs were downregulated in leukemia and lung cancer (**Figure 1A**). **Table 1** shows that mRNA expression levels of *FCGR1A/B*, *FCGR2A/B/C*, and *FCGR3B* were remarkably upregulated in ccRCC in multiple datasets. As shown in **Figure 1B**, eight FcγRs are expressed abnormally in different tumor tissues. mRNA expression levels of *FCGR1A/B/C*, *FCGR2A/B/C*, and *FCGR3A* were remarkably upregulated in ccRCC tissues compared with normal tissues. The protein expression levels of FcγRs were analyzed using the CPTAC online tool of UALCAN platform. It was observed that only *FCGR1A* expresses the functional FcγRI, whereas *FCGR1B/C* represents duplicated pseudogenes of *FCGR1A* (6). **Figure 1C** showed that the protein expression levels of *FCGR1A*, *FCGR2A/B*, and *FCGR3A* were downregulated in ccRCC tissues compared with normal tissues.

**Table 1:** Remarkable changes of FcγRs mRNA expression level between ccRCC and normal tissues (ONCOMINE).

	Types of PAAD vs. normal	Fold Change	t-test	P-value	
*FCGR1A*	ccRCC vs. Normal	3.623	9.036	4.57E−08	Gumz (27)
	ccRCC vs. Normal	2.856	6.211	0.0000179	Lenburg (28)
	Non-Hereditary ccRCC vs. Normal	7.552	8.972	5.46E−10	Beroukhim (29)
	Hereditary ccRCC vs. Normal	7.431	10.208	1.21E−09	Beroukhim (29)
	ccRCC vs. Normal	11.288	10.468	0.00000119	Yusenko (30)
*FCGR1B*	ccRCC vs. Normal	2.14	6.5	0.0000219	Lenburg (28)
	Non-Hereditary ccRCC vs. Normal	5.369	7.956	2.8E−09	Beroukhim (29)
	Hereditary ccRCC vs. Normal	5.47	9.463	1.35E−09	Beroukhim (29)
*FCGR2A*	ccRCC vs. Normal	2.829	8.636	4.35E−08	Gumz (27)
	ccRCC vs. Normal	2.261	4.97	0.0000706	Lenburg (28)
	Non-Hereditary ccRCC vs. Normal	4.659	8.597	0.000000215	Beroukhim (29)
	Hereditary ccRCC vs. Normal	6.143	10.413	4.63E−08	Beroukhim (29)
*FCGR2B*	ccRCC vs. Normal	5.212	4.849	0.0000777	Gumz (27)
	Hereditary ccRCC vs. Normal	3.466	6.29	0.00000059	Beroukhim (29)
	Non-Hereditary ccRCC vs. Normal	2.844	5.369	0.00000786	Beroukhim (29)
*FCGR2C*	Papillary Renal Cell Carcinoma vs. Normal	4.799	7.231	0.00000082	Yusenko (30)
	ccRCC vs. Normal	6.779	11.343	0.00000085	Yusenko (30)
	ccRCC vs. Normal	2.805	7.224	0.000000812	Gumz (27)
	Non-Hereditary ccRCC vs. Normal	3.15	7.08	0.000000799	Beroukhim (29)
	Hereditary ccRCC vs. Normal	4.139	9.021	0.00000006	Beroukhim (29)
*FCGR3B*	Non-Hereditary ccRCC vs. Normal	9.706	7.8	6.54E−09	Beroukhim (29)
	Hereditary ccRCC vs. Normal	15.915	11.751	8.03E−10	Beroukhim (29)
	ccRCC vs. Normal	9.204	4.699	0.0000895	Gumz (27)
	ccRCC vs. Normal	2.814	8.701	3.2E−09	Jones (31)

### Correlation Between Transcriptional Levels of FcγRs and Tumor Stages and Cancer Grade of ccRCC Patients

**Figure 2:**
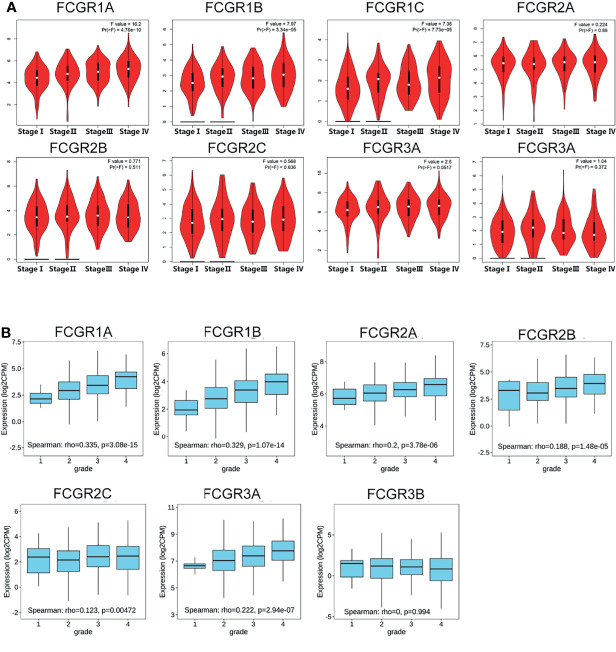
Correlation between FCgRs family expression and tumor stage/cancer grade in ccRCC patients. **(A)** mRNA expressions of FCGR1A/B/C and FCGR3A were significantly related to individual tumor stage (GEPIA) of patients, **(B)** mRNA levels of FCgRs except FCGR3B were associated with the individual cancer grade (TISIDB) of patients.

We used the GEPIA dataset to analyze the relationship between transcriptional levels of different FcγRs members with tumor stages of ccRCC patients. The results showed that the mRNA levels of *FCGR1A/B/C* and *FCGR3A* were correlated with the tumor stages of ccRCC patients, whereas the mRNA levels of *FCGR2A/B/C* and *FCGR3B* did not markedly differ among tumor stages (**Figure 2A**). The reason why the mRNA expression of FCGR2A/B/C and FCGR3B in ccRCC did not appear to be significantly different among tumor stages may be their unique roles in anti-tumor immunity. Likewise, cancer grades analysis by TISIDB indicated that mRNA expressions of *FCGR1A/B*, *FCGR2A/B/C*, and *FCGR3A* correlated with cancer grade of ccRCC (**Figure 2B**). In short, the results above suggested that mRNA expressions of FcγRs (except for *FCGR3B*) were positively correlated with individual tumor stages or cancer grades of patients.

### The Prognostic Value of FcγRs in Patients With ccRCC

**Figure 3:**
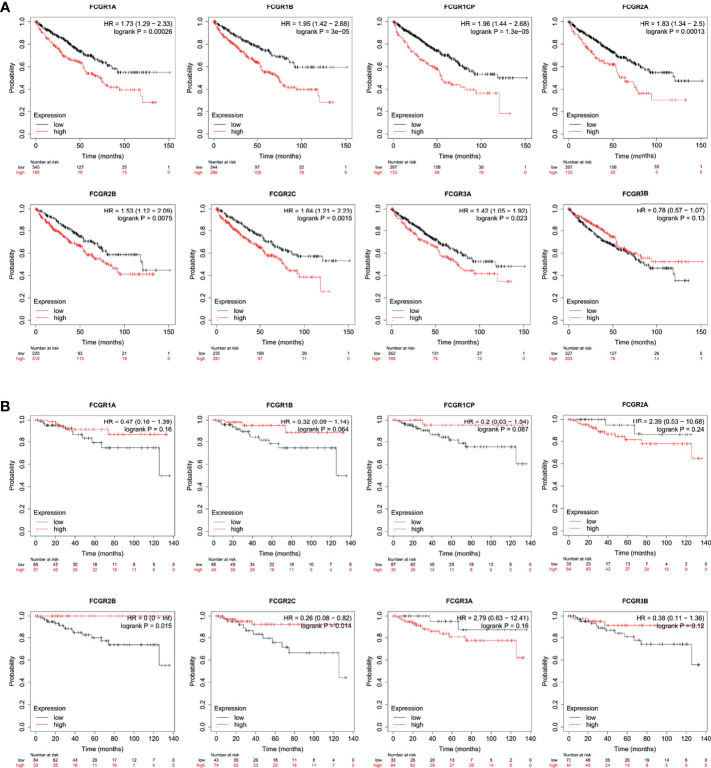
Prognostic feature of mRNA expression of distinct FCgRs in ccRCC patients (Kaplan–Meier plotter). The OS **(A)** and FPS** (B)** survival curves comparing patients with high (red) and low (black) FCgRs expression in ccRCC were plotted using the Kaplan–Meier plotter database at the threshold of p-value of <0.05.

To evaluate the prognostic value of the FCGR gene family in ccRCC progression, we analyze the correlation between FcγRs transcription levels and clinical outcomes including overall survival (OS) and disease-free survival (DFS) using the Kaplan–Meier Plotter database. ccRCC patients were divided into low and high-risk groups based on cutoff value. As shown in **Figure 3A**, high transcription levels of FcγRs were correlated with shorter OS in ccRCC. Nevertheless, high transcription levels of *FCGR2B/C* were correlated with longer DFS in ccRCC, and no significant correlation was observed between DFS and other FcγRs (**Figure 3B**). We downloaded and screened the gene expression and clinical data of 485 ccRCC patients from the TCGA database (**Supplementary Table 1**) for multivariate Cox regression survival analysis. The results showed that the effects of *FCGR1A*, *FCGR1B*, and *FCGR1C* on prognosis were still significant after correcting for conventional prognostic factors (**Table 2**; **Supplementary Table 2**).

**Table 2:** The Summary Results of Cox Regression Survival Analysis.

	Coefficient	Z_value	HR	Lower (95%)	Upper (95%)	P-value
*FCGR1A*	0.4544	2.7858	1.5753	1.1442	2.1687	0.0053
*FCGR1B*	0.5154	3.1135	1.6743	1.2104	2.316	0.0018
*FCGR1C*	0.7155	4.2799	2.0452	1.4738	2.8382	<0.0001
*FCGR2A*	0.2864	1.8138	1.3316	0.9772	1.8145	0.0697
*FCGR2B*	0.2243	1.4302	1.2514	0.9203	1.7017	0.1527
*FCGR2C*	0.2624	1.6728	1.3001	0.956	1.7681	0.0944
*FCGR3A*	0.1823	1.1595	1.2	0.8817	1.6331	0.2463
*FCGR3B*	-0.0413	-0.2594	0.9596	0.7025	1.3107	0.7954

### Correlation Between FcγRs DNA Methylation Levels and Clinical Outcomes in Patients With ccRCC

**Figure 4:**
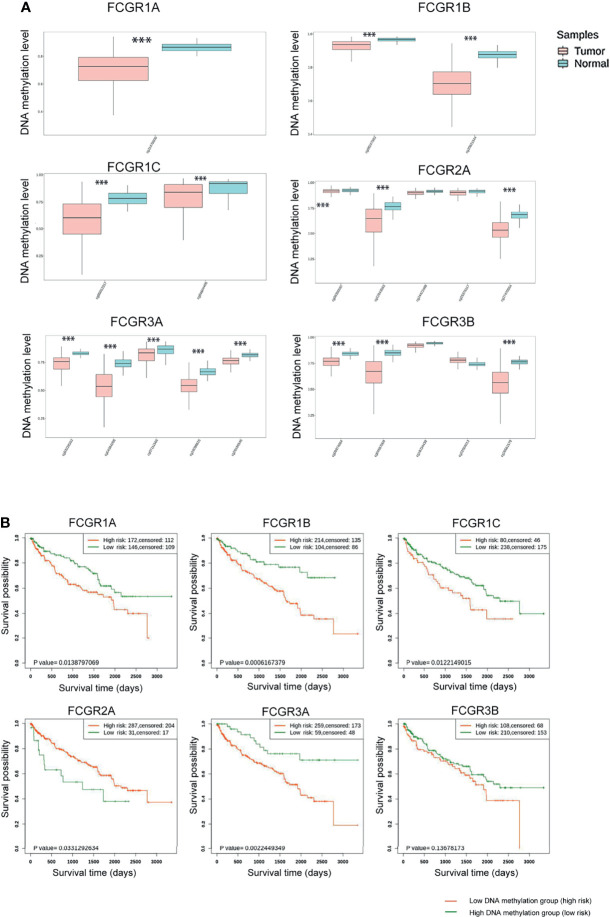
SurvivalMeth analysis of FCγRs. **(A)** FCγRs DNA methylation were enhanced in ccRCC tissues compared with normal renal tissues (***p <0.001). **(B)** The prognostic value of different FCγRs DNA methylation levels in ccRCC patients in the OS curve.

Genome-wide DNA methylation array and clinical outcome profiles of renal tissues were explored on the SurvivalMeth platform to investigate the DNA methylation levels of FcγRs and their relationships with clinical outcomes of ccRCC patients. Methylation levels of ccRCC were tested in Illumina Infnium HumanMethylation 450 array and Illumina Infnium HumanMethylation27 array in 535 tumors versus 357 normal renal tissues (318 tumors vs. 160 normal with HumanMethylation450 array; 217 tumors vs. 197 normal with HumanMethylation27 array). Lower DNA methylation levels of *FCGR1A/B/C*, *FCGR2A*, and *FCGR3A/B* were detected in ccRCC tissues, comparing with normal tissues (**Figure 4A**), whereas, the DNA methylation levels of *FCGR2A/B* did not differ significantly between tumors and normal tissues. Moreover, lower *FCGR1A/B/C* and *FCGR3A* DNA methylation levels were associated with shorter OS, while lower *FCGR2A* DNA methylation level was associated with longer OS (**Figure 4B**; **Table 3**).

**Table 3:** The Summary Results of Kaplan–Meier Plots.

	Concordance (CI)	Rsquare	HR	Lower (95%)	Upper (95%)	P-value
*FCGR1A*	0.551273	0.005437	1.660288	1.115112	2.471999	0.01388
*FCGR1B*	0.6050909	0.0510752	2.373975	1.5645872	3.6020732	0.0006167
*FCGR1C*	0.57315	0.01565	1.68973	1.06181	2.68898	0.01221
*FCGR2A*	0.58606	0.02623	0.54579	0.26666	1.11711	0.03313
*FCGR3A*	0.548061	0.017906	2.549054	1.60572	4.04658	0.002245
*FCGR3B*	0.612	0.04607	1.35641	0.8914	2.06402	0.13678

### PPI and Functional Enrichment Analysis of FcγRs and Their 88 Co-Expression Genes in ccRCC Patients

**Figure 5:**
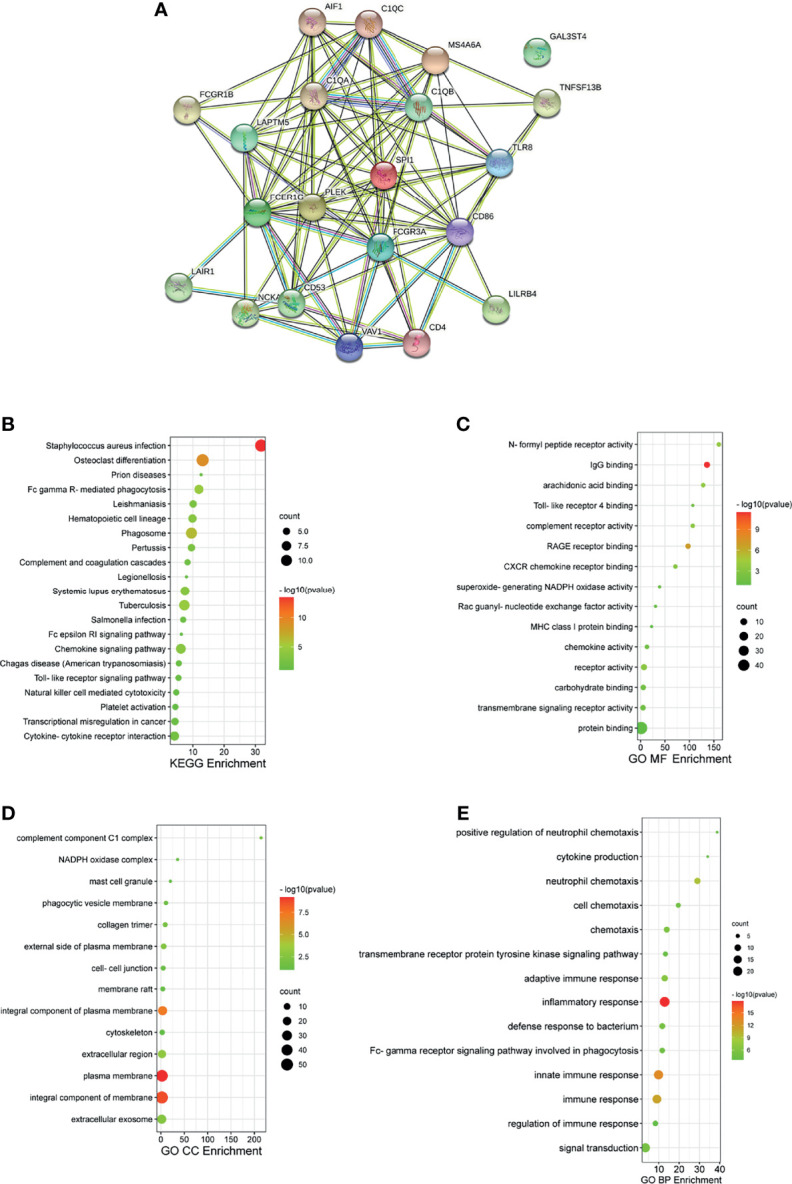
PPI and functional enrichment analysis of FCγRs and their 88 co-expression genes in ccRCC patients (STRING and DAVID). **(A)** PPI network. The nodes meant proteins; the edges meant the interaction of proteins **(B)** KEGG enriched terms. **(C)** GO MF enriched terms. **(D)** GO CC enriched terms. **(E)** GO BP enriched terms.

We then analyzed significant coexpression genes with FcγRs using the co-expression analysis module in the UAICAN database and listed in **Supplementary Table 3**. A total of 88 upregulated genes were significantly associated with FcγRs expression. Subsequently, the 88 genes were analyzed using GO and KEGG tools in DAVID, and constructed a PPI network by STRING. **Figure 5A** exposed that the activation of immune response-related genes, namely, C1QA, C1QB and, C1QC and adaptive immune response participant genes, such as LAIR1, LILRB4, CD4, and CD86 were closely connected with FcγRs alterations. The first 21 Kyoto Encyclopedia of Genes and Genomes (KEGG) pathways of FcγRs and their 88 Co-expression genes are illustrated in **Figure 5B**. Among them, Phagosom, FcγR-mediated phagocytosis, Cytokine–cytokine receptor interaction, Toll-like receptor signaling pathway and Natural killer cell mediated cytotoxicity are significantly associated with anti-tumor immunity of ccRCC. In addition, GO (Gene Ontology) analysis including molecular functions (MF), cellular components (CC), and biological processes (BP) are shown in **Figures 5C**–**E**. Most results of GO analysis were associated with immune responses.

### Correlation of FcγRs Expression Levels With Immune Infiltration in ccRCC

**Figure 6:**
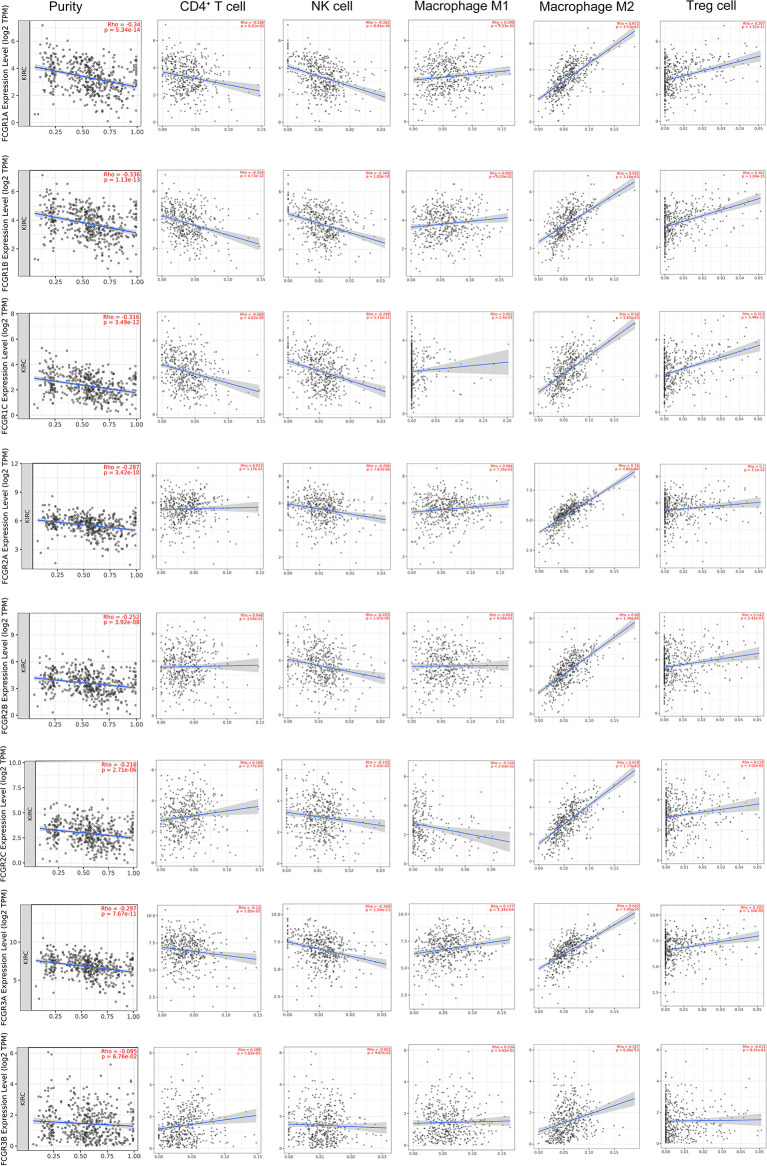
The relationship between FCγRs Expression Levels and Immune Infiltration Levels in ccRCC (TIMER). The correlation between the abundance of immune cell and the expression of FCγRs in ccRCC.

**Figure 7:**
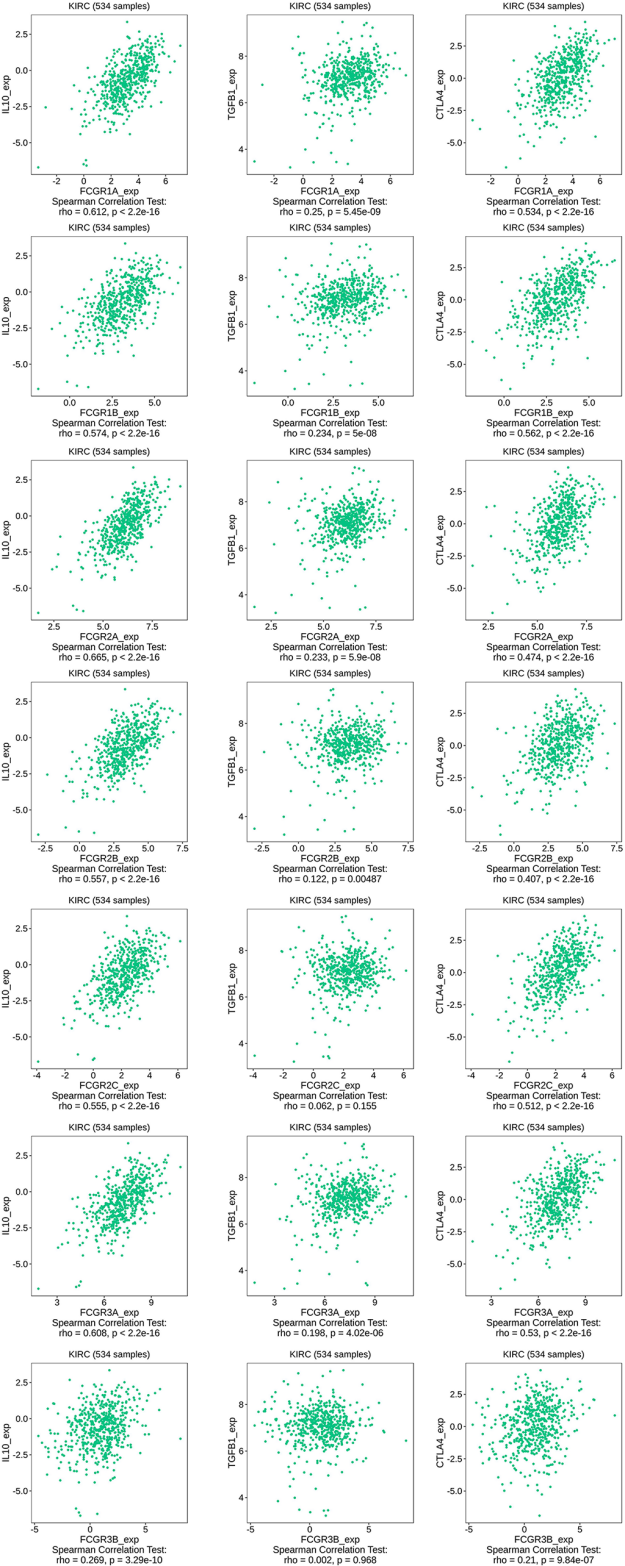
The relationship between FCγRs Expression Levels and Immunoinhibitor in ccRCC (TISIDB).

TIMER and TISIDB online analysis tools were used to evaluate the relationship between the expression levels of FcγRs and the level of immune infiltration in ccRCC. It was found that FcγRs are involved in immunosuppression regulation and immune cell infiltration, which might affect the clinical outcome of ccRCC patients. The analysis results showed that CD4^+^ T and NK were negatively correlated with FcγRs expression levels, whereas Treg and M2 macrophage cells were positively correlated with FcγRs expression levels (**Figure 6**). The result of the TISIDB online analysis shows that immunoinhibitors, namely, IL-10 and CTLA-4 were positively correlated with all the FcγRs expression levels. TGFB1 was positively correlated with *FCGR1A/B/C*, *FCGR2A/B*, and *FCGR3A* (**Figure 7**).

## Discussion

In the past few decades, monoclonal antibodies (mAbs) that directly target tumor cells have become powerful tools in the fight against cancer, by triggering elimination of cancer cells through FcγRs-mediated antibody-dependent cellular cytotoxicity (ADCC) or, phagocytosis (ADCP) and activating FcγRs on antigen-presenting cells (APC) to promote APC maturation (26). FcγRs were reported to be essential in anti-tumor immunity. FcγRI was demonstrated to play a central role in antibody therapy of experimental melanoma (32). DeLillo and Ravetch showed that the initial ADCC-mediated elimination of tumor cells is dependent on activating human FcgRIIIa using a murine model of EL4 lymphoma (33). The authors also demonstrated that the immune complex binding to FCγRIIa is an essential step in the activation of the T cell-dependent vaccinal effect. Indeed, patients carrying the allelic variants of *FCGR2A*, *FCGR2C*, and *FCGR3A* which exhibit increased affinity for human IgG demonstrated better responsiveness to anti-tumor antibody therapy in cases of B cell lymphomas, colorectal, renal, and breast cancers (20, 34–37).

Abnormal FcγRs expression was rarely reported in tumors. Only *FCGR2B* has been identified to be selectively expressed by metastasis melanoma that impairs the tumor susceptibility to FcγR-dependent innate effector responses, which might explain in part the low response of melanoma patients treated with anti-idiotype (38). In the present study, we found that all FcγRs members have remarkably high expression in ccRCC, and patients with higher FcγRs expression levels exhibit a worse prognosis. Among them, *FCGR1A/B/C* and *FCGR3A* more highly express in ccRCC. Then we analyzed the DNA methylation levels of the FcγRs and found that almost all FCGR genes have high methylation levels in ccRCC, and patients with higher methylation levels have a worse prognosis. The above results indicate that the low DNA methylation levels of the FcγRs in ccRCC were likely to decrease their transcription levels, which in turn affects the prognosis of the patient.

RCC is an extremely heterogeneous cancer, in which a complex immune microenvironment provides favorable conditions for tumor immune escape (39). RCC consists of three major histopathologic groups—ccRCC), papillary (pRCC), and chromophobe RCC (chRCC). Pan-RCC clustering according to RNA-sequencing data revealed a distinct histology-independent RCC subgroup characterized by strengthened mitochondrial and weakened angiogenesis-related gene signatures (40). RCC cells may induce cytokine expression, such as IL-10 and TGF-β, in the tumor microenvironment (TME), leading to an immunosuppressive tumor state and promoting immune escape (41–43). Tumor-related immunosuppressive cells, namely, regulatory T cells and tumor-associated macrophages, also play an “accomplice” role in the immunosuppressive tumor state (42). In the present study immune infiltration analysis showed that the expression of FcγRs was negatively correlated with infiltration levels of NK and macrophage M2 cells which were the major immune cells that eliminate tumor cells through ADCC or ADCP. Whereas the infiltration level of macrophage M1 and Treg cells was positively correlated with the expression of FcγRs which would contribute to the immunosuppressive state in ccRCC. The infiltration level of CD4^+^ T cells is negatively correlated with the expression levels of *FCGR1A/B/C* and *FCGR3A* and positively correlated with the expression of *FCGR2C*. NK cells and macrophages M1 are the primary cells that exert anti-tumor immunity through ADCC. High expression of FcγRs in tumor cells may competitively bind to anti-tumor monoclonal antibodies, thereby inhibiting the activation of ADCC, resulting in low infiltrate levels of NK cells in tumor tissues. Macrophages M2 and Treg cells play an immunosuppressive role in most tumor microenvironments, and the increased level of infiltration of both in ccRCC may lead to suppression of anti-tumor immunity, leading to a poor prognosis for patients. Further infiltration analysis of immune-related factors in the TISIDB online tool shows that immunosuppressive factors like IL-10, TGFB1, and CTLA-4 are positively related to FcγRs gene expression in ccRCC. In short, the increase of FcγRs expression level in ccRCC is likely to inhibit anti-tumor immune response by inhibiting the effect of ADCC and promoting the infiltration of immunosuppressive cells and immunosuppressive factors.

Emerging evidence indicates that angiogenesis and immunosuppression frequently occur simultaneously in tumor (44). Sasha et al. demonstrated that humanized or human IgG1 antibodies inhibited angiogenesis by binding to FcγRI of macrophages, resulting in reduced infiltration of macrophages in the tumor microenvironment (45). High expression of FCGR1 in ccRCC may compete with macrophages for binding to human IgG1 antibodies, thus inhibiting their antiangiogenic effects. The expression of FC**γ**Rs in ccRCC may simultaneously promote angiogenesis and immunosuppression.

To conclude, our research indicates that DNA methylation levels of FcγRs in ccRCC decreased and mRNA levels increased in ccRCC, which were both associated with poor clinical outcomes. FcγRs can be used as potential survival prognostic biomarkers and therapeutic target for ccRCC. The correlation between the expression of FcγRs and immune infiltration suggests that FcγRs may be involved in anti-tumor immunity in ccRCC. Our results indicated that FcγRs not only can be used as a risk factor for survival of patients with ccRCC but also reflect their immune status. Targeting the FcγRs might go a long way to find more appropriate prognostic factors for ccRCC as well as facilitate the development of novel immunotherapies.

## Data Availability Statement

The original contributions presented in the study are included in the article/**Supplementary Material**. Further inquiries can be directed to the corresponding author.

## Author Contributions

WN conceived the project and wrote the manuscript. SL, BL, JZ, and WL participated in data analysis. YY and TL participated in discussion and language editing. SY reviewed the manuscript. All authors listed have made a substantial, direct, and intellectual contribution to the work and approved it for publication.

## Conflict of Interest

The authors declare that the research was conducted in the absence of any commercial or financial relationships that could be construed as a potential conflict of interest.

## Publisher’s Note

All claims expressed in this article are solely those of the authors and do not necessarily represent those of their affiliated organizations, or those of the publisher, the editors and the reviewers. Any product that may be evaluated in this article, or claim that may be made by its manufacturer, is not guaranteed or endorsed by the publisher.

## Abbreviations

ccRCC, clear cell renal cell carcinoma; FcγRs, antibody-binding crystallizable fragment γ receptors; TCGA, The Cancer Genome Atlas; GO, Gene Ontology; KEGG, Kyoto Encyclopedia of Genes and Genomes; BP, biological processes; CC, cellular components; MF, molecular functions; OS, over survival; DFS, disease free survival.
